# Factors for thyroid autoimmunity in children and adolescents with type 1 diabetes mellitus

**DOI:** 10.3109/03009730903276381

**Published:** 2009-12-08

**Authors:** Kostas Kakleas, Evangelia Paschali, Nikos Kefalas, Aspasia Fotinou, Maria Kanariou, Christina Karayianni, Kyriaki Karavanaki

**Affiliations:** ^1^Diabetic Clinic, B’ Pediatric Department, University of Athens, ‘P. & A. Kyriakou’ Children’s Hospital, AthensGreece; ^2^Department of Immunology and Histocompatibility, ‘Aghia Sophia’ Children’s Hospital, AthensGreece; ^3^Department of Pediatric Endocrinology, ‘P. & A. Kyriakou’ Children’s Hospital, AthensGreece; ^4^Hormone Laboratory, ‘P. & A. Kyriakou’ Children’s Hospital, AthensGreece

**Keywords:** Childhood, subclinical autoimmune thyroiditis, thyroid autoantibodies, type 1 diabetes

## Abstract

**Introduction:**

Type 1 diabetes mellitus (T1DM) is associated with an autoimmune reaction to thyroid antigens including thyroid peroxidase (anti-TPO) and thyroglobulin (anti-Tg).

**Aims:**

We determined in children with T1DM the relationship of positive anti-thyroid antibodies to potential risk factors, including, age, gender, duration of diabetes, and glutamic acid decarboxylase antibodies (anti-GAD).

**Materials and methods:**

We studied 144 children and adolescents with T1DM. Their age was 12.3 ± 4.6 (mean ± SD) years, and duration of diabetes was 4.6 ± 3.8 years. Anti-thyroid antibodies were determined using a luminescence method and anti-GAD using an enzyme-linked immunosorbent assay.

**Results:**

The prevalence rates of anti-thyroid antibodies among the children with T1DM in our study were: anti-TPO (17.4%), anti-Tg (11.1%), and of both anti-thyroid antibodies (10.4%). The presence of serum anti-thyroid antibodies was positively associated with age (16.6 years in those with positive tests versus 12.0 years in those with negative tests, *P* = 0.027), duration of diabetes (7.4 versus 4.3 years, *P* = 0.031), and serum TSH (Thyroid-stimulating hormone) levels (4.8 versus 2.3 μIU/mL, *P* = 0.002). The presence of both anti-thyroid antibodies was associated with female sex (boys: 4/75 (5.3%), girls: 11/69 (15.9%), chi-square = 6.44, *P* = 0.04). Subclinical autoimmune thyroiditis (SAIT) was present in 55.5% of the patients with thyroid antibody-positivity and was positively associated with age (16.6 versus 12.0 years, *P* = 0.001) and diabetes duration (7.6 versus 4.2 years, *P* = 0.001). Multiple logistic regression analysis revealed that the development of anti-thyroid antibodies was predicted by: 1) the presence of anti-GAD (odds ratio (OR) 1.45, 95% confidence interval (CI) 1.09–1.92), 2) the presence of a second anti-thyroid antibody (OR 134.4, 95% CI 7.7–2350.3), and 3) older age (OR 22.9, 95% CI 1.13–463.2).

**Conclusions:**

Thyroid autoimmunity was associated with female gender, increasing age, long diabetes duration, the persistence of anti-GAD, and with TSH elevation, indicating subclinical hypothyroidism.

## Introduction

Children with type 1 diabetes mellitus (T1DM) are more prone to develop other organ-specific autoimmune diseases, among which autoimmune thyroiditis (AIT) is more frequently encountered (1–4).

The prevalence of thyroid autoimmunity in patients with T1DM has been reported to be two to four times more frequent than in the general population. Thus in adults the prevalence of positive anti-thyroid antibodies in the general population has been reported to be 6.6%–10% (5,6) and in Greece 13.9% (7), while in patients with T1DM it has been found to be higher, ranging from 20% to 40% (8,9).

Concerning children and adolescents, the relative prevalence in the general population was found to range from 2.9% to 3.4% (10,11), in Greece 4.6% (12), while in children and adolescents with T1DM it ranges from 19% to 23.4% (1–3). It is noteworthy that there is no relative study from Greece.

Different factors have been associated with the development of thyroid autoimmunity in the general population, such as heredity, increasing age, female gender, puberty, oestrogen use, pregnancy (12,13), and an iodine-rich diet (14,15). In adults with T1DM, female gender, increasing age, and the presence of glutamic acid decarboxylase antibodies (anti-GAD) have been associated with the development of thyroid autoimmunity (4,16). Also in children and adolescents with T1DM, previous studies agree on the age and gender effect (1,3,4,17,18), while there are very limited studies on the significance of the persistence of anti-GAD (4,16), the age at diabetes diagnosis (1,4), and diabetes duration (4,18,19) on the development of thyroid antibody positivity.

The development of autoimmune thyroiditis in children with T1DM has been associated with specific genetic risk markers. Specifically, hyperthyroidism has been related to the presence of HLA DQA1*0301, DQB1*0301, DQB1*0201, and hypothyroidism with HLA DQA1*0501 (18). The presence of DQB1*05 appears to be protective of the development of AIT (20).

AIT is characterized by the production of autoantibodies against the thyroid gland, T-lymphocytic infiltration of the gland, and subsequent development of various degrees of thyroid dysfunction (21). These autoantibodies are directed towards specific thyroid gland proteins, which are thyroglobulin (Tg), a fundamental component of thyroid colloid, and thyroid peroxidase (anti-TPO), an enzyme participating in the production of thyroid hormones (22).

The prevalence of anti-TPO in children with T1DM has been reported to be between 10% and 29.4% and that of anti-Tg between 8.7% and 14.4% (8,23–25), while the coexistence of both anti-thyroid antibodies has been reported in 5.9%–7% of the patients (8,24,25). These antibodies are not usually detected in the serum of T1DM children at diabetes diagnosis, but they seem to appear later on in the course of the diabetes in patients with the relative genetic predisposition (1,19). In a recent study, the presence of anti-thyroid antibodies at the onset of T1DM was detected in only 16.7% of the patients with thyroid autoimmunity (19).

Thus there are a limited number of studies thoroughly analysing the risk factors related to the development of thyroid antibodies in children with T1DM (4,19). It is noteworthy that there are also very few studies on the effect of thyroid antibody positivity on the growth and body mass index (BMI) status of children and adolescents with T1DM (26,27).

Therefore the aims of the present study were to identify, in Greek children and adolescents, the prevalence of thyroid antibody positivity and to determine the effect of potential risk factors, such as current age, age at onset of diabetes, duration of diabetes, and persistence of pancreatic autoimmunity (anti-GAD), on its development. Moreover we studied the possible effect of subclinical autoimmune thyroiditis on the growth and BMI status of children with T1DM.

## Patients and methods

The study population included 144 children and adolescents (male/female: 77/67) with T1DM, followed up in our out-patients’ diabetic clinic. The mean age (± SD) of the patients was 12.3 ± 4.6 years, with a mean age at T1DM diagnosis of 7.7 ± 3.6 years and a mean diabetes duration of 4.6 ± 3.9 years. The study was approved by the local Ethical Committee. Informed consent was obtained from each parent and/or patient before blood sampling.

The criteria for the diagnosis of T1DM diagnosis were: fasting plasma glucose levels of 126 mg/dL (7.0 mmol/L), or symptoms of hyperglycaemia (polyuria, polydipsia, and unexplained weight loss with a random plasma glucose ≥200 mg/dL (11.1 mmol/L), or 2-hour plasma glucose ≥200 mg/dL (11.1 mmol/L) during an oral glucose tolerance test (28). Apart from marked hyperglycaemia, the diagnosis of T1DM is usually associated with the presence of diabetic ketoacidocis (DKA) (29). Among our patients, 70.6% presented with DKA (pH < 7.30) and 29.4% without (pH ≥ 7.30). Thus 32.4% had severe DKA (pH < 7.10), 20.5% moderate (pH 7.10–7.19), and 17.7% mild DKA (pH 7.20–7.29). In the group without DKA (29.4%), ketones were present in the urine in 14% of the patients.

However there are additional serological markers of the autoimmune process that indicate and can predict T1DM, such as glutamic acid decarboxylase antibodies (anti-GAD) and islet antigen (IA-2) (30). Moreover c-peptide, an indicator of residual insulin production, is used for the differentiation between T1DM and T2DM (31). Among our patients, 66 (53.2%) were anti-GAD positive, while IA-2 and c-peptide levels were not routinely measured.

During each hospital visit, the patients were clinically examined, including thyroid gland palpitation, blood pressure measurement, and assessment of their pubertal status and growth. Thus height was expressed as height standard deviation scores (Ht-SDS) and body-weight as body mass index (BMI).

During the day of the data collection, venous blood was drawn cross-sectionally from each patient for autoantibody estimation, and sera were immediately stored at -20°C. Data on growth status and glycaemic control, as well as on previous autoantibody estimations, were recorded from the patients’ medical histories.

Enzyme-linked immunosorbent assay was used to detect anti-GAD antibodies (Euroimmun AG, Germany), performed with DYNEX DSX ELISA analyser. Isoform GAD65 from human recombinant glutamic acid decarboxylase was used. For the anti-GAD antibodies, the upper limit of the normal range was set at 10 IU/mL, and any greater value was considered as positive.

Anti-Tg and anti-TPO antibodies were detected using the luminescence method (ILMA, Nichols, Germany), performed with the Advantage analyser. The upper normal limit for anti-Tg antibodies was set at 100 IU/mL, while that for the anti-TPO antibodies was set at 16 IU/mL. Values greater than these cut-off values were considered as positive. For the evaluation of thyroid function, T4 (thyroxine), T3 (triiodothyronine), FT4 (Free-thyroxine), and FT3 (Free-triiodothyronine) levels were measured using the electrochemiluminescence method (CLIA) (Roche Diagnostics, France) on an ELECSUS 2010 analyser. Thyroid ultrasonography was performed in all patients with elevated titres of anti-thyroid antibodies, combined with thyroid enlargement and/or elevated TSH levels. The presence of thyroid enlargement (thyroid gland volume >97th age-related percentile) (32) with diffuse hypoechogenicity and/or diffuse micronodules confirmed the diagnosis of autoimmune thyroiditis (33). The diagnosis of subclinical autoimmune thyroiditis (SAIT) (Hashimoto’s) was based on high levels of TSH (>5 μIU/mL), associated with the presence of at least one thyroid autoantibody on two or more consecutive occasions, and/or with ultrasonographic findings of thyroiditis (33). Clinical hypothyroidism was associated, in addition to the above, with low FT4 levels and/or the presence of goitre. In all cases of hypothyroidism (subclinical or clinical) with positive anti-thyroid antibodies, with/without goitre, L-thyroxine administration was started.

Glycosylated haemoglobin A1c (HbA1c) was measured as an index of metabolic control on a DCA 2000 analyser. The normal range was 4.4%–6.4%.

### Statistical analysis

Data were analysed using SPSS version 13.0 (SPSS, Chicago, IL). The unpaired Student’s *t* test was performed for the comparison of means, and the chi-square test was used to compare percentages among different subgroups of patients. Multiple logistic regression analysis was used to assess the independency and strength of associations and to describe a predictive model.

## Results

Altogether 144 children and adolescents with T1DM (males/females: 75/69) were included in the present study, with a mean ± SD age of 12.35 ± 4.64 years (range 2.00–20.4), a mean ± SD age at diagnosis of diabetes of 7.74 ± 3.6 years (range 0.20–13.4), and a mean ± SD duration of diabetes of 4.66 ± 3.99 years (range 0.60–16.00). The mean ± SD HbA1c levels during the study period were 8.18% ± 1.68% (range 5.6%–14.4%), and the mean BMI of the patients was 20.60 ± 3.75 kg/m^2^ (range 13.6–35.09).

Among our patients, 66 (53.2%) were positive for anti-GAD antibodies and 26/144 (18.0%) for anti-thyroid antibodies; 25 of the 144 patients (17.4%) were positive for anti-TPO and 16/144 (11.1%) for anti-Tg; 10 of the patients (6.9%) were positive only for anti-TPO, and 2 of 144 (1.3%) were positive only for anti-Tg. A total of 15 (10.4%) patients were positive for both anti-TPO and anti-Tg.

In 4 of 27 patients (14.8%) tests for anti-thyroid antibodies were positive at the time of diagnosis of T1DM. Tests for anti-TPO antibodies became positive an average of 3.4 ± 3.5 (range 0–11) years after the diagnosis of T1DM. Tests for anti-Tg antibodies became positive 6.6 ± 3.5 years (range 1–13.0) after the diagnosis of T1DM. Tests for both anti-TPO and anti-Tg antibodies became positive 5.2 ± 2.9 years (range 0.4–10.0) after the diagnosis of T1DM.

### Factors contributing to the manifestation of thyroid autoimmunity

The prevalence of anti-thyroid antibodies was positively associated with diabetes duration (Table I) and age (Table II), with the highest prevalence rates observed in patients with a diabetes duration of ≥6 years (*P* = 0.014) or an age of ≥15 years.

**Table I.** Prevalence of positive autoantibodies in children with T1DM according to diabetes duration.

	Diabetes duration 0–2.9 years	Diabetes duration 3.0–5.9 years	Diabetes duration ≥6 years	*P*
	*n* = 65	*n* = 32	*n* = 46	
Anti-GAD antibodies:				
Anti-GAD(+)	61.5%	45.8%	47.5%	NS
Anti-thyroid antibodies:				
Anti-TPO(+)	9.2%	15.6%	30.4%	0.014
Anti-Tg(+)	3.1%	3.1%	28.3%	0.001
Anti-TPO(+) and anti-Tg(+)	3.0%	3.1%	26.0%	0.001

**Table II.** Prevalence of positive autoantibodies in children with T1DM according to current age.

	Current age 5–9.9 years	Current age 10–14.9 years	Current age ≥15 years	*P*
	*n* = 34	*n* = 62	*n* = 39	
Anti-GAD antibodies:				
Anti-GAD(+)	40.7%	62.3%	47.1%	NS
Anti-thyroid antibodies:				
Anti-TPO(+)	8.8%	11.3%	25.0%	0.033
Anti-Tg(+)	2.9%	6.5%	22.2%	0.001
Anti-TPO(+) and anti-Tg(+)	2.9%	6.4%	25.6%	0.002

A multiple logistic regression model was developed to analyse the effect of various factors, such as age, gender, age at T1DM diagnosis, diabetes duration, and the presence of pancreatic autoimmunity (anti-GAD) on the development of each thyroid antibody (Table III). Logistic regression analysis showed that the appearance of anti-Tg and anti-ΤPO antibodies was determined by age. Thus an increase of the age at diabetes diagnosis by 1 year increased the probability of thyroid antibody positivity by 15%. Moreover, a significant positive association between the prevalence of anti-Τg antibodies and current age was observed (odds ratio (OR) 22.9). Additionally, the presence of one type of anti-thyroid antibody increased the probability for the development of the other type of anti-thyroid antibody (OR 134.4). Finally the presence of anti-GAD was associated with a 2-fold greater risk for the development of anti-Tg (OR 1.45). However, given the effect of current age, age at diagnosis, and pancreatic autoimmunity, which were included in the logistic regression model, diabetes duration did not increase the probability of thyroid antibody positivity.

**Table III.** Logistic regression analysis for the estimation of the factors associated with autoantibody positivity in children with T1DM.

	*B*	*P*-value	OR	95% CI
Anti-Tg(+) versus Anti-Tg(-):				
Anti-TPO	4.90	0.001	134.4	(7.7–2350.3)
Current age (years)	0.37	0.041	22.9	(1.13–463.2)
Anti-GAD	3.13	0.01	1.45	(1.09–1.92)
Anti-TPO(+) or/and anti-Tg(+) versus anti-TPO(-) and anti-Tg(-):				
Age at diabetes diagnosis (years)	0.15	0.044	1.16	(1.00–1.35)

### Characteristics of patients with different types of anti-thyroid antibodies

We divided our study population into three groups according to the number of detected anti-thyroid antibodies. One group had negative tests for anti-thyroid antibodies, one group had one of two tests positive, and the third group had both tests positive for anti-thyroid antibodies (Table IV). The group of children with double thyroid antibody positivity were older and had a longer duration of diabetes than the other groups (*P* = 0.027 and *P* = 0.031 respectively). They also had elevated serum TSH concentrations (4.8 ± 1.6 μIU/L) but normal serum T4 levels (8.8 ± 1.1 mcg/dl), consistent with subclinical hypothyroidism. In contrast, serum TSH concentrations were lower (3.8 ± 1.9 μIU/L) in the group with only one of two positive tests. Females predominated in the group with double thyroid antibody positivity (boys 4/75 (5.3%), girls 11/69 (15.9%), chi-square = 6.44, *P* = 0.04).

**Table IV.** Characteristics of children with T1DM according to the presence or the absence of anti-thyroid antibodies.

	Absence of thyroid antibodies	One thyroid antibody positive (+)	Both thyroid antibodies positive (+)	*P*
	*n* = 117	*n* = 12	*n* = 15	
Current age (years)	12.0 ± 4.8	11.9 ± 5.6	16.6 ± 4.7	0.027
Age at diabetes diagnosis (years)	7.8 ± 3.8	8.2 ± 3.9	8.9 ± 4.7	NS
Males (*n* = 75)	62 (82.6%)	9 (12%)	4 (5.3%)	chi-square = 6.44
Females (*n* = 69)	55 (79.7%)	3 (4.3%)	11 (15.9%)	*P* = 0.04
Diabetes duration (years)	4.3 ± 3.7	3.8 ± 2.7	7.4 ± 4.5	0.031
BMI (kg/m^2^)	20.5 ± 3.5	20.9 ± 4.6	21.4 ± 4.9	NS
HtSDS	0.20 ± 1.21	0.22 ± 0.72	0.16 ± 1.0	NS
T4 (μg/dL)	9.2 ± 2.5	9.2 ± 1.1	8.8 ± 1.1	NS
TSH (μIU/L)	2.3 ± 0.8 ^a^	3.8 ± 1.9	4.8 ± 1.6 ^b^	NS

^a^ versus ^b^: *P* = 0.002.

### Thyroid status of children with anti-thyroid antibodies

At the time that anti-thyroid antibodies were first noted to be positive all patients were euthyroid with a mean age of 10.7 ± 4.24 years (range 3.2–19.0) and a mean diabetes duration of 3.5 ± 3.5 (range 0–11.0) years. After 3.1 ± 2.8 years (range 0–7 years), a progression towards subclinical hypothyroidism due to Hashimoto’s thyroiditis was observed in 15 of 27 (55.5%) patients. This diagnosis was based on the presence of elevated serum TSH concentrations (>5 μIU/mL) and normal T4 values, associated with a positive test for at least one anti-thyroid antibody, and/or with ultrasonographic findings of thyroiditis. Serum TSH levels in patients with subclinical hypothyroidism due to Hashimoto’s thyroiditis were 5.16 ± 2.6 μIU/L, and serum T4 levels were normal (8.8 ± 1.1 μg/dL), while no patient developed clinical hypothyroidism. Out of 27 patients 7 (25.9%) developed thyroid enlargement (goitre) together with anti-thyroid antibody positivity, and another 8 patients developed diffuse hypoechogenicity without thyroid enlargement. Patients with subclinical autoimmune thyroiditis (*n* = 15) were older (16.6 versus 12.0 years, *P* = 0.001) and had a longer diabetes duration (7.6 versus 4.2 years, *P* = 0.001) than the rest of the study population.

After the diagnosis of subclinical hypothyroidism, they received treatment with L-thyroxine at a dose of 100 μg/m^2^ of body surface area.

### Effect of subclinical autoimmune thyroiditis on the growth and BMI status of children with T1DM

No significant effect of anti-thyroid antibody positivity on the growth and BMI status of the children with diabetes was observed (Ht-SDS 0.062 versus 0.18, *P* = NS, BMI 21.7 versus 20.5 kg/m^2^, *P* = NS).

## Discussion

The present study reports on the prevalence of thyroid antibody positivity and of subclinical autoimmune thyroiditis in children and adolescents with T1DM in Greece and on the risk factors for its development. It is noteworthy that there is no relative previous study, to our knowledge, in Greece. Among the novelties of our study are the association of thyroid autoimmunity with the presence of anti-GAD, the effect of age at diabetes diagnosis and diabetes duration on the development of thyroid autoimmunity, the long-term follow-up of the patients and the progress from thyroid antibody positivity to subclinical and clinical hypothyroidism, and also the effect of subclinical hypothyroidism on the children’s growth. To our knowledge there are very limited studies in the literature on the above topics.

The prevalence rate of anti-thyroid antibodies in the T1DM patients of our study was 18.75%, while a significant percentage (55.5%) of them presented subclinical autoimmune thyroiditis relatively early in the course of the disease. Our findings are in agreement with previous studies in this age-group, reporting a prevalence of thyroid antibody positivity of 10%–23.4% (1,19,34), and of SAIT of 45% (1). It is worth mentioning that the prevalence rates of thyroid antibody positivity in adult T1DM patients are higher than the ones in children and adolescents and range from 20% to 40% (with the highest rates observed in middle-aged women) (35), while the prevalence of autoimmune thyroiditis in the general population fluctuates from 6.6% to 13.9% (5–7).

In our patients’ group we have noticed a significant association between the double thyroid antibody positivity and/or SAIT with female sex. Similar findings have been previously observed (25,36). Specifically, De Block et al. (36) reported a 3-fold risk of anti-TPO antibody positivity in female adolescents and young adults with diabetes in comparison with males. Also in the general population, girls are more prone to develop thyroid disease than boys (12). Actually, sex hormones have been reported to affect the development of antibodies (36). In patients with T1DM (37) and also in an animal model of autoimmune thyroiditis (38), oestradiol seemed to accelerate the progression of autoimmune diseases via enhancing the pathway of T helper type 2 (Th2) cells, while androgens had a protective effect (39).

In this study, the prevalence of thyroid antibodies increased with increasing age and diabetes duration. Our findings are in agreement with previous studies (1,36), reporting that the highest prevalence of thyroid antibodies was observed after the age of 15 years, or after a diabetes duration of 3.5 years. This observation suggests that autoimmune disease is the final phase of a process starting with autorecognition, passing through immunity with the appearance of autoantibodies, and finally leading to cell destruction and autoimmune disease (39). Moreover it is known that the maximum autoimmune activity is observed during puberty (39). Actually in our study, although the role of diabetes duration in the development of thyroid antibody positivity was found to be significant in the univariate analysis, in the multivariate analysis it was eliminated, given the effect of age. This could be explained by the fact that in adolescent girls, apart from diabetes duration, the presence of female hormones may significantly contribute to the development of thyroid autoimmunity.

Another interesting finding of the present study was that there was no significant effect of subclinical autoimmune hypothyroiditis on growth and BMI status in T1DM patients, which is in agreement with certain studies (1,27). However, Chase et al. (26) reported reduced growth rates in children with T1DM and subclinical hypothyroidism, particularly in those with TSH levels ≥10 μIU/L, while thyroid hormone replacement therapy led to improved growth only in prepubertal patients. Thus the clinical importance of the early detection and treatment of SAIT in children and adolescents with T1DM could be the prevention of growth impairment. In terms of the BMI status of children with diabetes and thyroid autoimmunity, no significant effect was observed in our study, which is in agreement with previous studies (22,24).

We also observed an increase of TSH levels, directly proportional to the degree of anti-thyroid antibody positivity, with the lowest values occurring in the group without thyroid autoimmunity and the highest ones in the group with double thyroid antibody positivity. A possible explanation for this observation could be that in the presence of both thyroid antibodies, the immune stimulation is probably more intense, resulting in thyroid dysfunction. However, it is not known whether these organ-specific autoantibodies are directly involved in the pathophysiologic mechanism of thyroid gland destruction or whether they are associated with tissue destruction by thyroid-infiltrating T cells (40). In previous studies, anti-TPO antibodies seemed to be more specific markers of thyroid gland function, as patients with positive anti-TPO antibodies had higher TSH levels than those with positive anti-Tg antibodies (1,41). In the present study, TSH levels did not significantly differ between the above two groups of patients.

An important observation of our study was the association of thyroid autoimmunity with the persistence of pancreatic autoimmunity. This finding is in agreement with another study (4), reporting that T1DM patients with anti-GAD positivity had a 2-fold greater risk for the development of thyroid autoimmunity than those without anti-GAD. A possible explanation for this association could be that anti-GAD antibodies are not exclusively present in the brain and pancreas but can also be found in other tissues, such as the follicle cells of the thyroid gland and also the parietal cells of the stomach (42,43). Thus the persistence of anti-GAD antibodies could be a marker for the future development of autoimmunity against the thyroid gland and other organs.

In conclusion, the presence of thyroid antibody positivity and the subsequent development of subclinical autoimmune thyroiditis were quite prevalent among the children and adolescents with T1DM of our study, while the possible risk factors for its development were older age ≥15 years, female gender, long diabetes duration, and the persistence of anti-GAD. Subclinical hypothyroidism was not found to affect the children’s growth and BMI status. Thus, it is suggested that all patients with T1DM should be screened for autoimmune thyroiditis upon diagnosis and then yearly, and in case of thyroid antibody positivity they should be regularly followed up in terms of their thyroid function and growth status.
